# Jeannie N. Shinozuka, *Biotic Borders: Transpacific Plant and Insect Migration and the Rise of Anti-Asian Racism in America, 1890–1950*, Chicago: University of Chicago Press, 2022, 296 pp

**DOI:** 10.1007/s10739-023-09759-z

**Published:** 2023-12-05

**Authors:** Lisa Onaga

**Affiliations:** Max Planck Institute for the History of Science, Berlin, Germany

*Biotic Borders* reconstructs the history of the formation of ideas of race by examining the consequences of human, plant, and insect movements across the Pacific Ocean and North America between the late 19th century and the 1940s. Jeannie Shinozuka shows that US anxieties about the end of the colonial frontier and limitations of resources led white settler colonists to seek out and import exotic, that is, “transpacific” plants (p. 9), which invited a cascade of events that simultaneously led to the racialization of plants, along with their diseases, insect pests, and human plant propagators. Shinozuka’s book introduces a new perspective toward the analysis of sources concerning the history of entomology in the US, especially in the context of the institutionalizing field of economic entomology in the USDA and Federal Horticultural Board. The book accomplishes this by detailing the development of US settler colonial scientific responses to the pests and diseases associated with plants shipped from Asia alongside analyses of a rich trove of published sources, manuscripts, as well as oral histories normally limited to Asian American studies scholarship. In doing so, this revisionist history of gardens, floriculture, and horticulture shows the historical emergence of a discernible pattern, wherein different tripartite units of plants, insects, and humans became contentiously interconnected in the policing and deliberation of national borders. The book contributes a long-awaited historical analysis of the interrelationship of science and the production of fear that surrounded the dehumanization of people of Asian-Pacific descent during the early decades of the 20th century, which was integral to the expansion of nativist sentiments and policies in the US.

Through a series of chapters organized around key institutional and disciplinary developments and controversial cases of plant introductions, *Biotic Borders* shows how local US demand for exotic plants and developments in floriculture and horticulture spurred new problems with the spread of new insects and plant diseases, which generated quarantine and other control measures. The book opens by establishing how the US in the 1890s had regarded Asian humans as a new post-slavery era pool of cheap labor, establishing a tension in the narrative between a focus on the actions of government researchers and the experiences of Asian Americans whose opportunities were limited to work such as gardening, agriculture, and fishing. The book proceeds by relating the history of plant canker control to the geopolitics of new plant quarantine laws that foreshadowed controversial human immigration policies. The task of classifying the origins of “dangerous insects,” including the blowback of Japanese entomological efforts to internationalize and cooperate on the ecological guardianship of national borders, is shown to have only stoked racialization. Discussion of efforts to control pests such as the San José scale and the characterization of Japanese gardeners as unhygienic “little brown men” illustrate the gradual rendering of both Japanese migrants and Japanese American citizens into aliens to be feared (p. 105–106).

By focusing particularly on Japanese immigrants in North and South America, Mexico, and Hawai‘i (before it became part of the Union in 1959), the book compellingly reveals the making and displacement of responsibility and agency when it came to regulating plants, insects, and diseases and handling immigrants that enabled the mass incarceration of people of Japanese descent in the Americas during World War II. Chapters on the prophylactic and biocontrol measures against the Japanese beetle in Hawai‘i and pesticide use in the East Coast (to protect exotic plants naturalized in Philadelphia’s nature trails) show how derogatory names that dehumanized Japanese people developed around racializing rhetoric used to market the efficacy of chemical interventions. The penultimate chapter turns to the human even more directly. It explores the exclusionist rhetoric within “survival of the fittest” discourse by tracing the construction of fear stemming from biological characterizations of mixed-race offspring of Japanese and white Americans as aberrant (despite the known advantages of plant hybridization), reinforcing the discourse of the degeneration of races (p. 167). While the history of eugenics in the US has been well-examined, Shinozuka illuminates how eugenic thought had solidified in the country with practical plant exploration, showing how the continual comparison of Japanese plants with those on American soil reinforced how these biological beings were only welcome so long as they remained useful or pleasing––while opening the path to racially scientizing Japanese people and their height as degenerate, due to their plant-based diets. The final chapter examines how white fishers and vegetable growers leveraged public fears to eliminate their economic competition, namely by permitting the growth of accusations that Japanese American fishers and growers were deliberately poisoning the public with over-use of pesticides, contrary to scientific screenings that showed otherwise. Shinozuka argues that the subsequent wartime incarceration of Japanese Americans highlighted their civil rights violations in a way such that it rendered them nonthreatening to the “native biota,” ironically referring to descendants of white settler colonialism (p. 199).

This history joins conversations in the history of science that have reflected upon the experiences of Indigenous American communities in the Northern and Southern Hemispheres. *Biotic Borders* uniquely models a reparative way to narrate a complex history of dispossession by referring to plants, insects, and humans as immigrants. Shinozuka’s daring method may be unnerving to readers accustomed to analyzing the works of biologists and other scholars who avoid anthropomorphizing nonhumans. In her commitment to describing plants and their symbionts as immigrants, Shinozuka directs nuanced attention to the naturalization processes of Japanese plants in the American landscape, including the Orientalization that selectively invited curiosity and interest in exotic plants while turning away from the problems that were generated consequently or compounded by negatively characterizing human beings associated with their points of origin or care. Recategorizing travelers from “more-than-human” worlds (as the book occasionally phrases) as “immigrants” (pp. 11, 129, 141) seems to strategically draw critical attention to the normalized way historians have continued to talk about insects, diseases, and humans as invasive and foreign. All in all, *Biotic Borders* provides a detailed, systematic analysis of how Asian immigrants became ensnarled historically with practices of pest control. This eye-opening work will be required reading for updated historical understandings about the relationship between biological science, racism, and nationalism.

